# Asthma Severity and Prevalence: An Ongoing Interaction between Exposure, Hygiene, and Lifestyle

**DOI:** 10.1371/journal.pmed.0020034

**Published:** 2005-02-22

**Authors:** Thomas A. E Platts-Mills

## Abstract

Why are the prevalence and severity of asthma increasing? Platts-Mills looks at the key studies that can help to anwer this important question

Over the last hundred years, there have been major triumphs in medicine related to public health, vaccination, and the introduction of new medicines. However, over the same period, several diseases have increased in prevalence and/or severity. In some cases, the causes of the increase have become obvious—the increases in lung cancer, coronary artery disease and type 2 diabetes, for example, are not considered to be a mystery. On the other hand, a large group of diseases related broadly to “inflammation” have also increased. For these, a wide range of hypotheses about causation have been proposed. Type 1 diabetes, rheumatoid arthritis, and inflammatory bowel disease have increased since 1980 [1]. Some analyses of the increase in hay fever and asthma would suggest a similar time course, and this parallelism of the time frame has been taken to suggest that there could be a common cause. Indeed, there is a proposal that these diseases are all related to some changes in “cleanliness” or “hygiene” that have resulted in decreased activation of a common control mechanism. Specifically, this control has been ascribed to T regulator cells, which produce interleukin-10 (IL-10) or transforming growth factor-beta.

## Change in Living Conditions

The major changes in rural areas, tropical villages or in Europe pre-1900 that could be related to the change in immune responses include: decreases in helminth infection; physical proximity to farm animals [2]; exposure to those mycobacteria that are commonly found in the soil; bifidobacteria colonization of the gut; as well as decreased prevalence of Hepatitis A infection [3]. The implication in each case is that asthma has increased secondary to an increase in inflammation or an increase of the allergic response that is closely associated. This assumes that hay fever, asthma, and other diseases have increased in parallel, which is probably not true.

Hay fever became a problem in Northern Germany and England as early as 1900. Clear evidence for this view comes from Noon's description of the development of immunotherapy for hay fever in 1911, the studies on hay fever prevalence by Ratner and Silverman in New York in 1935, and the recognition that hay fever was a major community problem in New York in 1946. More recently, Harold Nelson analyzed all the studies on hay fever published in the United States and found a prevalence of ~15% in 1960, with no convincing evidence of an increase since then (H. Nelson, personal communication).

If seasonal hay fever was epidemic in 1960, then the subsequent increase in asthma has to be seen in a different light. The best estimates for the start of the asthma epidemic are around 1960 for such diverse populations as army recruits in Finland [4], school children in Birmingham in the United Kingdom, and African American children in Charleston, South Carolina, United States [5]. In each of these studies, the increase in asthma symptoms or disease has been greater than tenfold. However, the absolute values of the change have been dramatically different in New Zealand and Scotland (from ~2% up to 20%) compared with Finland (from 0.2% to 4%) [6]. Furthermore, some countries have experienced much greater increases in hospitalization than others. Clear evidence for increases in mortality has only come from the United Kingdom, New Zealand, and the United States, countries with a high prevalence of symptoms and hospitalization.

Several hypotheses have been proposed to explain the increase in asthma (Box 1). At this point, our questions about the increase in asthma are: 1) Is an increase in allergy or hay fever a necessary precursor for the increase in asthma, a parallel event, or separate? 2) Why did the increase in asthma have such a consistent time course throughout the Western world in countries where changes in infectious diseases have occurred very differently? and 3) Why is asthma more common and more severe in some countries than in other countries that have had an equal scale of increase (i.e., tenfold) over the same time course?

**Box 1. Hypotheses about the Cause of the Increase in Asthma: Arguments For and Against**

**Hypothesis 1: Increased exposure to perennial allergens, e.g., dust mites**

**For:**

(A) Housing changes: houses are built more tightly and are more well insulated; more furnishings; fitted carpets, and (B) more time spent indoors. Increased exposure leads to increased sensitization.

**Against:** Increases in asthma have been seen in countries where dust mites are not present in homes, and in the Netherlands the concentration of mite allergens has actually decreased by as much as tenfold over the past 15 years

**Hypothesis 2: Changed immune responsiveness is due to cleanliness **

**For:**

(A) Bacterial and other infections have decreased due to improved hygiene, immunization, and antibiotics, and (B) changed gut flora (antibiotics, diet, etc.). Change from Th1 to Th2 leads to increased allergy.

**Against:** In New York City, the increase in seasonal hay fever occurred 30 years before the increase in asthma, and in Africa, children whose families move into cities, including informal settlements, have experienced increases in infections, wheezing, and diagnosed asthma

**Hypothesis 3: Loss of a lung-specific protective effect, 1960–2000**

**For:**

(A) Changing diet leads to a changed inflammatory response, and (B) decline in physical exercise. Increased wheezing among allergic children.

**Against:** This is unlikely to be just be a lung-specific effect

## The Persistent Association between Specific IgE, Total IgE, and Asthma

**Figure 1:**
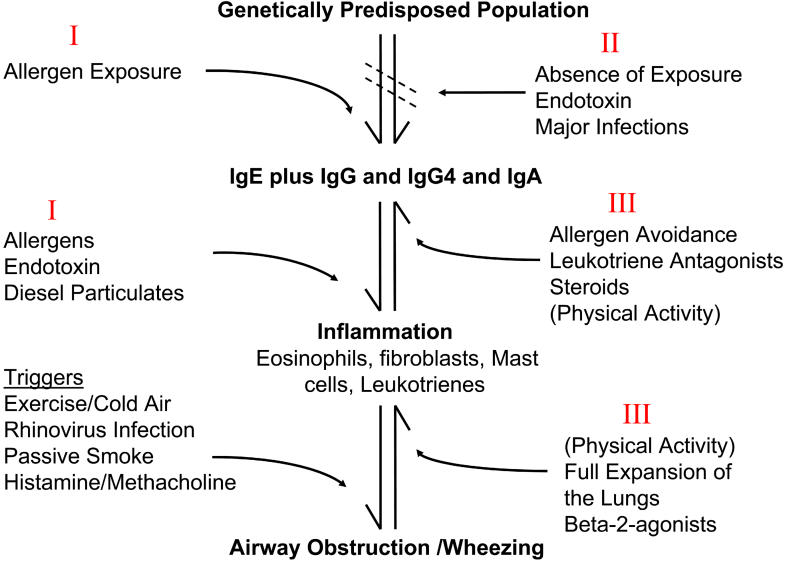
Sensitization, Inflammation, and Wheezing Increases in prevalence/severity of asthma (reversible airway obstruction) could occur because of changes in different parts of the hypersensitivity and inflammatory response in the lungs. The Roman numerals refer to the three hypotheses in Box 1.

Long before the first case control or prospective study, the association between allergy and asthma was obvious in case series. These earlier studies reported skin testing with an amorphous extract called “house dust,” but it was not until the identification of dust mites that the association was clarified [7]. Indeed, as late as 1978, there were significant doubts that allergens played a role in asthma. Using extracts of Dermatophagoides pteronyssinus (dust mites), the strong association between this allergen and asthma was established in many parts of the world, with odds ratios as high as 6 or even 10 [7,8]. The possibility of a causal relationship was further supported by bronchial challenge studies and avoidance experiments [9]. The cohort in Poole, Dorset was, until recently, the only study with household measurements of allergen and the results strongly suggested that exposure in the child's own house was the primary determinant of sensitization [8]. Subsequently, studies from other parts of the world provided evidence about other indoor allergens, particularly cats, dogs, and the German cockroach [10,11]. These studies showed that perennial exposure to allergens was an important cause of inflammation in the lungs and associated nonspecific bronchial hyperreactivity (Figure 1). In most of the case-control and prospective studies, sensitization to seasonal pollens has not been significantly associated with asthma [7]. This is an important issue; if allergy is associated with asthma for genetic reasons or because of some common immunological feature, it is not clear why the association should be with perennial allergens only.

Since assays for total serum IgE became available in the 1970s, it has been clear that patients with asthma have, on average, higher total IgE than patients with hay fever or no allergy. Indeed, by 1980 this was considered an established fact in textbooks of immunology. It was assumed that the increased total IgE related to allergen-specific responses. However, in some studies, the association between total IgE and asthma was stronger than the association between asthma and specific IgE. In 1989, Burrows et al. went further and suggested that specific IgE correlated with hay fever, while total IgE correlated with asthma [12]. The implication was that IgE has a complex relationship with asthma that is not dependent on specific allergens.

The strength of the association between asthma and total IgE raises questions that have not been resolved. Do specific IgE antibody responses contribute to or even push total IgE? If so, do the IgE antibody responses to some allergens have more effect than others? This question is relevant both to attempts to explain major differences in total IgE between countries and to studies on acute asthma. In emergency room and hospital studies, the geometric mean total serum IgE of patients with asthma is often greater than 200 IU/ml higher than values found in population-based studies. Recent work from Heymann et al. and Green et al. on patients hospitalized for asthma has suggested that the interaction between rhinovirus and allergy occurs predominantly among patients with total IgE > 200 IU/ml [13]. Thus, the different properties of allergens could influence both the prevalence and severity of asthma. However, the properties of the dominant allergens do not explain the overall increase in prevalence, which has occurred in countries with very different houses, climates, and traditions of domestic pet ownership.

## The Paradoxical Relationship between Cat Ownership and Sensitization: Significance for Prevalence or Severity

Antigen exposure is considered to be a primary requisite for immune responses, and allergen-specific responses are no exception. There are many examples of allergens that are not significant in areas where the allergen is not encountered. For example, the pollen of olive trees is not relevant in northern countries, dust mite allergens are not significant in the northern part of Scandinavia or the mountain states of the United States, and cockroach allergens are not significant in suburban areas of the United States, the United Kingdom, or New Zealand.

**Figure 2:**
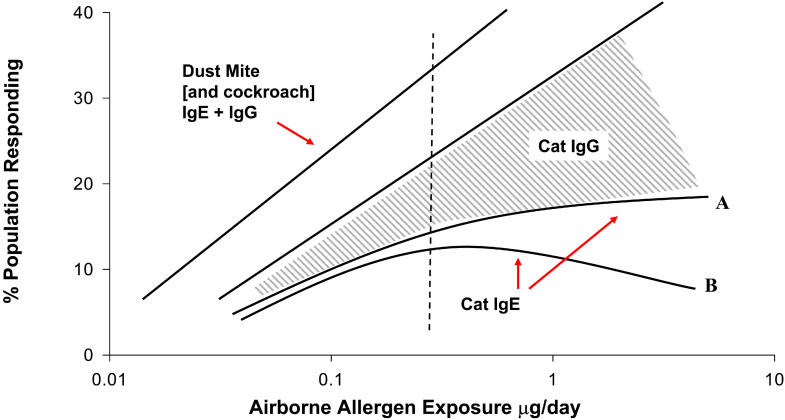
Contrast between Exposure to Dust Mite or Cat Allergens and the Relevant Immune Responses The dashed line indicates the approximate value of 20 mg Fel d 1/g floor dust or the presence of a cat.

For dust mites, there is a wide range of evidence that increased exposure increases sensitization. Homes in Sweden, Berlin, the United Kingdom, and New Zealand have progressively higher concentrations of mite allergen and progressively higher prevalence of sensitization to mite allergens [7]. But there is now evidence that increasing exposure to cats does not lead to a higher prevalence of allergy [14,15,16]. On a population basis, the effect may be profound; sensitization to cats among school-age children is generally ~10%, while mite sensitization is often as high as 30% (Figure 2). This effect cannot be ascribed to inadequate exposure, since all estimates of the quantity of cat allergen inhaled are higher than for dust mites. Furthermore, the quantity of cat allergen found in schools or even in houses without cats is sufficient to sensitize at-risk children [11].

**Figure 3:**
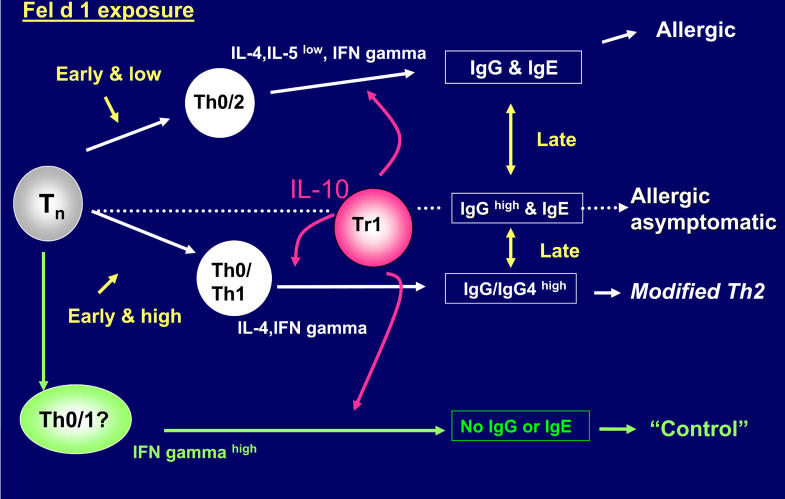
Mechanisms of T Cell Control over the Hypersensitivity Response to Cat Allergen The response to cat allergen, Fel d 1, includes T cells that produce high levels of IL-10 that have some of the features of the T regulator one cells (Tr1). However, these cells appear to be a feature of both allergic and non-allergic responses, implying that the whole response to cat allergen is controlled. Some of the effects of cat exposure may need to occur early, but other studies suggest strongly that the different responses can change following a prolonged change in exposure late in life.

In fact, children raised in a house with a cat were less likely to be sensitized to cats [15]. Initially, it seemed possible that the effect was an example of reverse causation. However, the effect has been observed in countries where a large proportion of families keep cats, and very few families report choosing not to own a cat because of asthma in the family. Furthermore, the presence of a cat in a house in New Zealand does not decrease IgE antibody response to dust mites—in other words, tolerance to cats can be cat-specific [17]. Understanding how the response to cat allergen is controlled could provide an insight into how both the prevalence and the titre of IgE antibody responses in general are (or should be) controlled. It seems inevitable that the primary control is by T cells specific for cat allergens. Indeed, there is already excellent evidence that injection of peptides derived from the cat allergen Fel d 1, which give T cell responses, can be used for immunotherapy [18]. Studying overlapping peptides of Fel d 1, we identified a striking response to two peptides at the terminal end of Chain 2. Furthermore, both allergic and tolerant individuals respond to these peptides by producing IL-10 and interferon gamma [19]. The implication is that Fel d 1 inherently induces control, and that this control influences both allergic and non-allergic responses to cat allergen (Figure 3). In keeping with this, IgE antibody responses to cat allergen are not quantitatively as high as those to dust mites [17].

One of the central assumptions of the cleanliness hypothesis is that regulation of immune responses is, at least in part, non-specific. It is assumed that helminth infection, mycobacteria, Hepatitis A, endotoxin, and early-life infections can create a milieu that leads to a decrease in allergic responses in general. Likewise, we might expect that exposure to high concentrations of cat allergen, which can induce IL-10 producing cells, should have a general effect. There is some evidence for this hypothesis. In Detroit, the presence of two or more animals in the house tended to reduce allergy in general. In Sweden, the presence of a cat in the house is associated with decreased sensitization to cat, and also to birch and dog. However, in the United States and New Zealand, the presence of a cat in the home has no effect on the prevalence or titre of IgE antibody to dust mites [15,17]. Thus, it is clear that under some circumstances the tolerance to cats can occur in highly atopic individuals and be cat-specific. This phenomenon is not in keeping with any version of the cleanliness hypothesis. It has been proposed that animals in the home could have a non-specific effect because they shed or encourage endotoxin. However, recent studies on airborne endotoxin found that the presence of cats had no effect, and that there were significantly lower endotoxin levels in houses with cats compared to dogs.

## The Possible Role of Lifestyle Changes

Over the last half of the 20th century, there have been major changes in diet and physical activity. The most obvious result of these changes is an increase in obesity, which has reached epidemic proportions in the United States. Since 1994, there have been multiple reports of an association between elevated body mass index (BMI) and asthma [20]. In 1996, we suggested that changes in physical activity could be related to bronchospasm [21]. Thus, there are three distinct but strongly interrelated aspects of lifestyle that could be relevant to the prevalence and severity of asthma: diet, physical activity, and obesity.

It is much easier to document BMI than diet or physical activity, and although some of the obesity data are convincing, they are not consistent and certainly not comparable to the association between obesity and diseases such as type 2 diabetes in childhood. In a typical study, Camargo et al. found that the prevalence of wheezing was 13% among the heaviest quintile and 7% among the lowest quintile for BMI [22]. Comparable values for type 2 diabetes would be 5% and 0.1%.

Studies using questionnaires have attempted to ask whether there is a relationship between wheezing and physical activity. However, the track record for questionnaires on this subject is very poor. Westerterp and his colleagues have reported two observations: first, that general activity contributes more to energy consumption than “aerobic exercise” does and second, that many subjects who initiate an exercise program (such as a twice weekly visit to the gym) overcompensate so that they actually decrease overall activity [23]. Recently, we have documented a decrease in activity among children (age ~4 years) in the United States Head Start program (a child-development program that aims to increase the school-readiness of young children in low-income families) who have a history of wheezing [24]. Although there are several possible explanations for this result, it seems clear that decreased activity can be present before elevated BMI.

The next question to ask is whether physical activity would have a beneficial effect on asthma? Fredburg concluded that full expansion of the lungs had a more potent effect on bronchial smooth muscle than isoprenaline [25]. Togias and his colleagues have shown that prolonged shallow breathing (=20 minutes) can lead to increased non-specific bronchial reactivity [26]. It is obvious that expansion of the lungs is decreased during prolonged periods of sitting down. However, periodic expansion of the lungs occurs with sighs. Recent data from our group shows that “sigh rates” while seated are very variable but are significantly lower when watching a video than while reading [27]. Thus there is a real possibility that some forms of childhood behavior—TV, videos, computer games, etc.—might be associated with sigh rates low enough to increase non-specific bronchial hyperreactivity.

A different explanation for the effects of physical activity comes from evidence that physical activity can be “anti-inflammatory.” This evidence relates to several different models though not at present to the lungs. In some studies, obesity appears to be a risk factor for wheezing among non-allergic children. However, in most studies, the association between allergen sensitization and asthma has been found in obese and non-obese children equally [28]. Obviously, some obese individuals are unfit and become breathless on exercise. In addition, these individuals may have sleep-disordered breathing. Thus, there are other conditions that are easily confused with exercise-induced asthma or nocturnal asthma. Taken together, there are excellent reasons for asking whether lifestyle changes have contributed to the increased prevalence or severity of asthma. However, it seems unlikely that this effect occurs on normal lungs, so the hypothesis has to be that decreased physical activity in patients who are allergic can allow persistent or increased severity of wheezing.

## Conclusions

Although the explanation for the increase in asthma is not yet clear, it is possible to put forward a model that includes elements of each of the three main hypotheses. Children raised in the tropics, on farms, or in villages such as those in Africa or Papua New Guinea have exposure to endotoxin or infections sufficient to interfere with the development of allergen-specific IgE antibody responses. Once water supplies are clean, and major infectious diseases have been controlled, allergic diseases will appear.

However, asthma appears to be associated with perennial, i.e., indoor exposure, and may be more common or more severe in countries where mites or cockroaches are the major source of allergens. Even with indoor allergen exposure, wheezing may remain transient or mild, provided prolonged outdoor play is normal. It is the combination of the control of infectious diseases, prolonged indoor exposure, and a sedentary lifestyle that is the key to the asthma epidemic and, in particular, the key to the rise in severity. Using this analysis, the severity of asthma in North American cities becomes much easier to explain. Children in New York, Atlanta, Philadelphia, and Washington, D.C. spend long hours indoors, have high exposure to mite, cockroach, and/or rodent allergens, and have very low levels of physical activity.

In conclusion, it appears that these combined factors are the key to the asthma epidemic and, in particular, the key to the rise in severity. We clearly need to develop ways to increase prolonged physical activity, both among patients with asthma and in the general population. We also need to investigate whether prolonged moderate activity is beneficial in the treatment of asthma and/or is “anti-inflammatory.” What is equally clear is that the current obsession of the medical profession with the pharmaceutical management of asthma (as well as other lifestyle-related diseases) does not address the reasons why the disease has become so common and so severe.

## Abbreviations

BMI: body mass index
IL-10: interleukin-10
